# An Experimental Pipeline for Initial Characterization of Bacterial Type III Secretion System Inhibitor Mode of Action Using Enteropathogenic *Yersinia*

**DOI:** 10.3389/fcimb.2018.00404

**Published:** 2018-11-22

**Authors:** Jessica M. Morgan, Hanh N. Lam, Jocelyn Delgado, Justin Luu, Sina Mohammadi, Ralph R. Isberg, Helen Wang, Victoria Auerbuch

**Affiliations:** ^1^Department of Chemistry and Biochemistry, University of California, Santa Cruz, Santa Cruz, CA, United States; ^2^Department of Microbiology and Environmental Toxicology, University of California, Santa Cruz, Santa Cruz, CA, United States; ^3^Department of Molecular Biology and Microbiology, Tufts University School of Medicine, Boston, MA, United States; ^4^Department of Medical Biochemistry and Microbiology, Uppsala University, Uppsala, Sweden

**Keywords:** type III secretion system, T3SS, T3SS inhibitor, *Yersinia*, pYV

## Abstract

Dozens of Gram negative pathogens use one or more type III secretion systems (T3SS) to disarm host defenses or occupy a beneficial niche during infection of a host organism. While the T3SS represents an attractive drug target and dozens of compounds with T3SS inhibitory activity have been identified, few T3SS inhibitors have been validated and mode of action determined. One issue is the lack of standardized orthogonal assays following high throughput screening. Using a training set of commercially available compounds previously shown to possess T3SS inhibitory activity, we demonstrate the utility of an experiment pipeline comprised of six distinct assays to assess the stages of type III secretion impacted: T3SS gene copy number, T3SS gene expression, T3SS basal body and needle assembly, secretion of cargo through the T3SS, and translocation of T3SS effector proteins into host cells. We used enteropathogenic *Yersinia* as the workhorse T3SS-expressing model organisms for this experimental pipeline, as *Yersinia* is sensitive to all T3SS inhibitors we tested, including those active against other T3SS-expressing pathogens. We find that this experimental pipeline is capable of rapidly distinguishing between T3SS inhibitors that interrupt the process of type III secretion at different points in T3SS assembly and function. For example, our data suggests that Compound 3, a malic diamide, blocks either activity of the assembled T3SS or alters the structure of the T3SS in a way that blocks T3SS cargo secretion but not antibody recognition of the T3SS needle. In contrast, our data predicts that Compound 4, a haloid-containing sulfonamidobenzamide, disrupts T3SS needle subunit secretion or assembly. Furthermore, we suggest that misregulation of copy number control of the pYV virulence plasmid, which encodes the *Yersinia* T3SS, should be considered as a possible mode of action for compounds with T3SS inhibitory activity against *Yersinia*.

## Introduction

The type III secretion system (T3SS) is a macromolecular nanosyringe used by dozens of Gram negative pathogens, including *Yersinia, Shigella, Salmonella, Chlamydia*, and *Pseudomonas*, to inject effector proteins into target host cells (Deng et al., 2017). The core structure of the T3SS consists of a basal body that anchors the entire complex in the bacterial membrane and assembles first. The basal body is composed of three proteins, which oligomerize into rings. SctD and SctJ form two rings in the bacterial inner membrane (IM) and SctC, a member of the secretin family of proteins, forms a ring in the bacterial outer membrane (OM) (Bergeron et al., 2013). These three rings are connected in the center by a hollow inner rod and the IM rings are closely associated with the SctN ATPase complex whose integrity is essential to the T3SS (Diepold et al., 2010). In addition to ATP, the proton motive force is also important for T3SS activity (Wilharm et al., 2004), although how the T3SS harnesses the proton motive force remains unclear. Once active, the basal body secretes the SctF needle protein, which polymerizes into a straight, hollow tube protruding into the extracellular space. In *Yersinia* species pathogenic to mammals, the fully assembled Ysc T3SS needle is composed of ~140 SctF subunits, is 65 nm in length, and harbors a tip complex composed of a pentamer of the hydrophilic LcrV translocator protein (Broz et al., 2007). Upon host cell contact, two additional hydrophobic translocator proteins, YopD and YopB, are secreted through the Ysc needle to form a translocon complex that leads to pore formation in the host membrane, facilitating the translocation of effector proteins to the host cytoplasm (Büttner and Bonas, 2002).

The *Yersinia* Ysc T3SS is highly regulated at the transcriptional, translational, and post-translational levels (Francis et al., 2002; Heroven et al., 2012). The transcription factor LcrF directs transcription of genes encoding the T3SS structural, regulatory, and effector proteins, all of which are encoded on the 70 kb pYV virulence plasmid (Schwiesow et al., 2015). Several factors govern regulation of LcrF expression, including temperature and the transcription factor IscR (Schwiesow et al., 2015). Importantly, pYV copy number increases during active type III secretion, and this is important for *Yersinia* virulence (Wang et al., 2016). In addition, the T3SS functions on a positive feedback loop in which active secretion leads to upregulated transcription of T3SS genes (Cornelis et al., 1987; Francis et al., 2002), although the mechanism behind this remains unclear.

A number of pathogens require one or more T3SSs for virulence, as genetic ablation causes attenuation in animal models and clinical isolates harbor plasmids or pathogenicity islands that encode T3SS genes (Coburn et al., 2007). An antibody against the *Pseudomonas aeruginosa* T3SS needle tip protein PcrV is part of a current Phase II clinical trial to treat nosocomial ventilator-associated pneumonia (NCT02696902), indicating that antibodies targeting the T3SS may be used as therapeutics. However, antibodies have low oral bioavailability and must be administered by injection; small molecules with high oral bioavailability are more attractive as therapeutic agents. A number of putative small molecule T3SS inhibitors have been identified in the past 15 years (Duncan et al., 2012; Marshall and Finlay, 2014; Anantharajah et al., 2017), yet only one class of compounds can be considered validated. Many published T3SS inhibitors have off target effects that may underlie their T3SS disruption. For example, the best studied class of T3SS inhibitors, the salicylidene acylhydrazides, are thought to cause deregulation of T3SS genes through an unknown mechanism, yet the activity of some salicylidene acylhydrazides is dependent on iron chelation (Beckham and Roe, 2014). The phenoxyacetamides represent the only class of compounds that inhibit the T3SS in a physiologically relevant cellular context, protect against a bacterial infection (*P. aeruginosa* abscess formation in mice), and have a validated molecular target, the SctF needle subunit (Bowlin et al., 2014; Berube et al., 2017).

We have developed an experimental pipeline that can be employed to determine initial mode of action for compounds with T3SS inhibitory activity. We chose to use the enteropathogens *Yersinia pseudotuberculosis* and *Yersinia enterocolitica* as the workhorses for this assay pipeline because *Yersinia* is susceptible to the majority of T3SS inhibitors described and because of the wealth of genetic and biochemical tools available. In addition, *Yersinia* are extracellular pathogens that use their T3SS to prevent phagocytosis, negating the need for a T3SS inhibitor to cross the mammalian cell membrane to inhibit the T3SS-host cell interaction. Importantly, the assays selected for the experimental pipeline had to be amenable to miniaturization, as compound availability is often limiting. Since interfering with T3SS gene expression, basal body assembly, needle assembly, and host cell effector protein translocation could all lead to inhibition of T3SS activity, we designed our pipeline to consist of distinct assays that each measure a specific stage of type III secretion. Inhibitors of LcrF and its homolog ExsA from *P. aeruginosa* have been described (Marsden et al., 2015). In addition, it is possible that a small molecule with T3SS inhibitory activity could exert its effect by inhibiting secretion-associated increase of pYV copy number, leading to a decrease in T3SS gene expression. Therefore, two pipeline assays measure pYV copy number and T3SS gene expression. Once T3SS genes are expressed, the T3SS basal body assembles followed by the T3SS needle. Two additional pipeline assays measure SctD (YscD in *Yersinia*) localization to the IM and SctF (YscF) needle assembly. The last two pipeline assays monitor efficiency of T3SS effector protein (Yop) secretion *in vitro* or Yop translocation into target host cells. Each individual assay on its own provides a limited snapshot of a compound's T3SS inhibitory activity. However, when performed as a pipeline of assays, the resulting data can be used to predict the mode of action of a T3SS inhibitor. Furthermore, the training set of T3SS inhibitors we use to validate this pipeline are commercially available, and therefore can serve as controls to compare the activity of other T3SS inhibitors.

## Materials and methods

### Compounds and antibodies

Compound 3 (CAS# 443329-02-0), compound 4 (CAS# 138323-28-1), and INP0007 (CAS# 300668-15-9) were obtained from Chembridge. INP0010 (CAS# 68639-26-9) was obtained from ChemDiv. Compound 20 (CAS# 489402-27-9) was obtained from TimTek. The anti-YscF antibody was raised in rabbits against the *Y. pseudotuberculosis* YscF peptide KDKPDNPALLADLQH (Morgan et al., 2017). DMSO concentration did not exceed 0.2%, except where indicated.

### Bacterial strains and growth conditions

Bacterial strains used in this paper are listed in Table 1. *Y. pseudotuberculosis* and *Y. enterocolitica* were grown, unless otherwise specified, in 2xYT (yeast extract-tryptone) at 26°C shaking overnight. In order to induce the T3SS, overnight cultures were diluted into low calcium medium (2xYT plus 20 mM sodium oxalate and 20 mM MgCl_2_) to an optical density (OD_600_) of 0.2 and grown for 1.5 h at 26°C shaking followed by 2–3 h at 37°C to induce Yop synthesis, depending on the assay, as previously described (Auerbuch et al., 2009).

**Table 1:** Bacterial strains used in this study.

**Strain**	**Description**	**References**
Wild type	*Y. pseudotuberculosis* IP2666, naturally lacking YopT expression	Bliska et al., 1991
Δyop6 pYopM-Bla	*Y. pseudotuberculosis* IP2666 Δ*yopHEMOJ* pYopM-Bla	Duncan et al., 2014
Δyop6/Δ*yop*B pYopM-Bla	*Y. pseudotuberculosis* IP2666 Δ*yopHEMOJ*/Δ*yopB* pYopM-Bla	Duncan et al., 2014
pYV40-EGFP-*yscD*	*Y. enterocolitica* serotype O9 strain E40	Diepold et al., 2010
Wild type	*Y. enterocolitica* 8081 serotype O8	Portnoy et al., 1981
YpIII/pIBX	*Y. pseudotuberculosis* YpIII with Tn5*luxCDABE* inserted in the Tn1000 resolvase homolog in pCD1 (wild type); Km^r^	Fahlgren et al., 2014
YpIII/(pIBX)_n = 1_	*Y. pseudotuberculosis* YpIII/pIBX derivative with the pIBX virulence plasmid lacking 3426 bp encoding the IncFII replicon and R6K suicide plasmid, integrated into YPK_3687 in the chromosome; Km^r^, Cm^r^	Wang et al., 2016
p*yop*H FLAG mCherry	*Y. pseudotuberculosis* YPIII containing the *yopH* promoter and YopH with an N-terminal FLAG tag and a C-terminal ssrA-tagged mCherry cloned into pMMB67EH	This study
*flhDC^*Y*.*pestis*^*	*Y. pseudotuberculosis* Δ*yopHEMOJ/flhDC*^*Y.*pestis**^	Auerbuch et al., 2009

### Construction of YopH transcriptional reporter

An expression cassette containing the *yopH* promoter and FLAG (5′ terminus)- and ssrA (3′ terminus)-tagged mCherry was generated using SOEing PCR and cloned into pMMB67EH (Horton et al., 1990; Pettersson et al., 1996; Karzai et al., 2000). The *yopH* promoter was amplified from pHYopT (gift from J. Bliska) using oligonucleotides SMP425 and SMP426 (Table 2). mCherry (gift from R. Tsien) was amplified using oligonucleotides SMP427 and SMP431. FLAG and ssrA sequences were incorporated into oligonucleotides SMP426/SMP427 and 431, respectively. Oligonucleotides SMP425 and SMP431 were used to generate the pyopH-FLAG-mCherry-ssrA cassette. PCR product was digested with BamHI and EcoRI and cloned into similarly digested pMMB67EH. Site directed mutagenesis using oligonucleotides SMP437 and SMP438 was used to generate the AAV variant of the ssrA tag.

**Table 2:** Oligonucleotides used to generate pYopH FLAG mCherry.

**Number**	**Sequence**	**Dir**	**Function**	**Enzyme**
SMP425	GGAggatccGCTGCGCGATGTACTGACCCG	>>	pyopH 5′	BamHI
SMP426	CTCCTCGCCCTTGCTCACCATCTTGTCATCGTCGTCCTTGTAATCCATATGTCCCTCCTTAATTAAATACACGCCTATAC	< <	pyopH-FLAG-mCherry/EGFP (for SOE, 3′, use with SMP425)
SMP427	GTATAGGCGTGTATTTAATTAAGGAGGGACATatggattacaaggacgacgatgacaagAtggtgagcaagggcgaggag	>>	pyopH-FLAG-mCherry/EGFP (for SOE, 5′, use with SMP428)
SMP428	GAAgaattcTTACTTGTACAGCTCGTCCATGCCG	< <	mCherry/EGFP 3′	EcoRI
SMP431	GAAgaattc*TTA*CGCTGCTAACGCGTAATTCTCATCATTCGCTGCCTTGTACAGCTCGTCCATGCCGC	< <	mCherry/EGFP 3′ with ssrA (LAA) tag (use with 425,427)	EcoRI
SMP435	CAGCGAATGATGAGAATTACGCGTTAGTAGCGTAAGAATTCTGTTTCCTGTGTG	>>	LAA->LVA mut. for pMMB67EH-PyopH-FLAG-mCherry (top)
SMP436	CACACAGGAAACAGAATTCTTACGCTACTAACGCGTAATTCTCATCATTCGCTG	< <	LAA->LVA mut. for pMMB67EH-pyopH-FLAG-mCherry (bottom)
SMP437	GCAGCGAATGATGAGAATTACGCGgCAGCAGTGTAAGAATTCTGTTTCCTGTGTGAAATTG	>>	LAA->AAV mut. for pMMB67EH-pyopH-FLAG-mCherry (top)
SMP438	CAATTTCACACAGGAAACAGAATTCTTACACTGCTGCCGCGTAATTCTCATCATTCGCTGC	< <	LAA->AAV mut. for pMMB67EH-PyopH-FLAG-mCherry (bottom)
SMP455	CAAGGCAGCGAATGATGAGAATTACGCGGCAtCAGtGTAAGAATTCTGTTTCCTGTGTGAAATTGTTATC	>>	LAA->ASV mut. for pMMB67EH-pyopH-FLAG-mCherry (top)
SMP456	GATAACAATTTCACACAGGAAACAGAATTCTTACACTGATGCCGCGTAATTCTCATCATTCGCTGCCTTG	< <	LAA->ASV mut. for pMMB67EH-pyopH-FLAG-mCherry (bottom)

### Type III secretion assay

Visualization of *Yersinia* T3SS cargo secreted in broth culture by Coomassie staining of SDS-PAGE separated proteins was performed as previously described with some modifications (Auerbuch et al., 2009). After low calcium medium cultures were grown for 1.5 h at 26°C, compounds or DMSO were added and the cultures shifted to 37°C for another 2 h. Post-incubation cultures were spun down at 15,000 × g for 10 min at room temperature. Supernatants were transferred to a new eppendorf tube. Ten percent final trichloroacetic acid (TCA) was added and the mixture was vortexed vigorously. Samples were incubated on ice for 20 min and then spun down at 15,000 × g for 15 min at 4°C. The pellet was resuspended in final sample buffer (FSB) with 20% DTT. Samples were boiled for 15 min prior to running on a 12.5% SDS-PAGE gel. Sample loading was normalized for bacterial culture density (OD_600_) measured prior to centrifugation. Densitometric quantification of the bands was done using Image Lab software (Bio-Rad), setting the relevant DMSO-treated YopE band to 1.00.

The miniaturized secretion assay was done by using *Y. pseudotuberculosis* expressing a YopM-β-lactamase (YopM-Bla) reporter and the chromogenic β-lactamase substrate nitrocefin to detect secreted YopM-Bla (O'Callaghan et al., 1972; Lee et al., 2007; Green et al., 2016). *Y. pseudotuberculosis* Δyop6 pYopM-Bla and Δ*yscNU* pYopM-Bla (negative control) were grown overnight at 26°C with 250 rpm shaking in 2xYT supplemented with chloramphenicol (25 μg/ml). The bacterial culture was diluted in low calcium media to OD_600_ 0.2, incubated at 26°C with 250 rpm shaking for 1.5 h, and 25 μl was distributed into a 384-well plate (Nunc^TM^, Thermo Fisher). Fifty micromolars of compounds or equivalent volume of DMSO was added to each well and the plate incubated at 37°C with 250 rpm shaking for 2 h. The bacterial plate was spun down for 5 min and 10 μl of supernatant transferred to a new plate containing the same volume of nitrocefin (500 μg/ml). Absorbance at 490 nm was measured 30 min after addition of nitrocefin. Three technical replicates for each sample were carried out for each independent experiment.

### Quantitative real-time PCR

Indicated concentrations of compounds, or the equivalent volumes of DMSO, were added to 26°C-grown low calcium medium cultures and treated cultures were shifted to 37°C for 3 h. RNA was isolated using an RNeasy Plus Micro Kit (Qiagen) according to the manufacturer's instructions and 2 μg RNA was used to make cDNA, as previously described (Auerbuch et al., 2009; Miller et al., 2014). SYBR Green PCR master mix (Applied Biosystems) was used for qPCR reactions according to the manufacturer's instructions and a 60°C annealing temperature, using the 16s rRNA gene as a reference for each sample. Three technical replicates were averaged for each sample/primer pair per independent experiment. Primers used are listed in Table 3. Results were analyzed using the Bio-Rad CFX software.

**Table 3:** qPCR primers used in this study.

**Name**	**Sequence**	**References**
FqyopE	CCATAAACCGGTGGTGAC	Morgan et al., 2017
RqyopE	CTTGGCATTGAGTGATACTG	Morgan et al., 2017
FqyscN	CTTCGCTTATTCGTAGTGCT	Miller et al., 2014
RqyscN	TCGCCTAAATCAGACTCAAT	Miller et al., 2014
Fq16s	AGCCAGCGGACCACATAAAG	Merriam et al., 1997
Rq16s	AGTTGCAGACTCCAATCCGG	Merriam et al., 1997
FqyscF	TCTCTGGATTTACGAAAGGA	Miller et al., 2014
RqyscF	GCTTATCTTTCAATGCTGCT	Miller et al., 2014
FqlcrF	GGAGTGATTTTCCGTCAGTA	Miller et al., 2014
RqlcrF	CTCCATAAATTTTTGCAACC	Miller et al., 2014
FqyscD	TGCCAGAGACGTTACAGGTT	This study
RqyscD	CATCCTGGTTATACTCGCGC	This study
FqErpA	TACCGGTGGTGGATGTAGCGGG	Miller et al., 2014
RqErpA	ATAATCCACGGCACCGCCCAC	Miller et al., 2014
FqyopK	ATGTTGCCATTCGTATAAGC	This study
RqyopK	GAGAACGGATGTTTGTTCAT	This study
FqyopH	ACACTACAAGACGCCAAAG	This study
RqyopH	GTGAAGGGCTGAATGTGAA	This study
FqlcrV	TGATATCGAATTGCTCAAGA	This study
RqlcrV	CGGCGGTTAAAGAGAAAT	This study
FqL9	TGGGTGACCAAGTCAACGTA	This study
RqL9	GTCGCGAGTACCGATAGAGC	This study

### YopH transcriptional reporter assay

Indicated concentrations of compounds, or the equivalent volumes of DMSO, were added to 26°C-grown low calcium medium cultures and treated cultures were shifted to 37°C for 3 h. Two-hundred microliters of cultures were spun down at 3,000 × g for 5 min, resuspended in 200 μl 1X PBS, and mCherry fluorescence and optical density measured in black, clear bottom 96 well plates (Costar®, Corning Inc.) on a Perkin Elmer Victor X3 plate reader. Two technical replicates were averaged for each sample per independent experiment.

### Immunofluorescence staining of YscF

Quantification of YscF staining on the bacterial surface was carried out as described previously (Morgan et al., 2017). Indicated concentrations of compounds, or the equivalent volumes of DMSO, were added to 26°C-grown low calcium medium cultures and treated cultures were shifted to 37°C for 3 h. Bacteria were fixed by the addition of a mix of 800 μl 4% paraformaldehyde, 1 μl 25% glutaraldehyde, and 40 μl 0.5 M NaPO_4_ pH 7.4 to 500 μl of bacterial culture for 15 min at room temperature followed by 30 min or longer on ice. Fixed bacteria were gently sedimented (5,000 × g for 5 min), washed four times with PBS and stored in 250 mM glucose, 10 mM Tris-HCl pH 7.5, and 1 mM EDTA. Eight microliters of the fixed cells were added to coverslips and allowed to set until just dried. Coverslips were blocked overnight with PBST with 3% BSA (PBST/BSA) at 4°C. Blocking solution was removed and anti-YscF primary antibody was added at 1:10,000 in PBST/BSA and rocked at 4°C for 4 h. Coverslips were carefully rinsed in ice cold PBST with 0.1% Tween-20 several times and incubated with Alexa fluor 594 or 488 anti-rabbit secondary antibody (Invitrogen) at 1:10,000 in PBST/BSA and rocked at 4°C for 3 h followed by rinsing again in ice cold PBST with 0.1% Tween-20 several times. Coverslips were then stained for nuclear material with Hoechst 33342 (Thermo Scientific) at 1:10,000 in PBST/BSA and left in the dark at room temperature for 30 min. Coverslips were washed in ice cold PBST with 0.1% Tween-20 several times, allowed to dry briefly, mounted onto glass coverslips with Prolong Gold (Thermo Scientific), and sealed with clear nail polish. Images were taken with the Zeiss Axioimager Z2 widefield microscope under 63X/1.4 oil immersion using Zen software, pseudocolored, and merged in FIJI. YscF puncta were counted using IMARIS software.

### YscD analysis

*Y. enterocolitica* expressing EGFP-YscD were grown overnight in brain heart infusion (BHI) broth containing nalidixic acid (35 μg/ml) and diaminopimelic acid (80 μg/ml) (Diepold et al., 2010). Overnight cultures were diluted to an OD_600_ of 0.2 in M9 minimal medium supplemented with casamino acids. Cultures were grown for 1.5 h at 26°C. Compounds were added at indicated concentrations or the equivalent volumes of DMSO were added and the cultures switched to 37°C for another 3 h. Two microliters of culture were layered on a patch of 1% agarose in water (Skinner et al., 2013) supplemented with 80 μg/ml diaminopimelic acid, 5 mM EDTA, 10 mM MgCl_2_, and either compounds at indicated concentrations or the equivalent volume of DMSO (Diepold et al., 2010). Images were taken with a Zeiss Axioimager Z2 widefield microscope under 63X/1.4 oil immersion using Zen software, pseudocolored, and merged in FIJI. YscD puncta were counted using IMARIS software.

### pYV copy number

*Y. pseudotuberculosis* YpIII/pIBX_N = 1_ and YpIII/pIBX were grown overnight in 2xYT media containing kanamycin (30 μg/ml). Overnight cultures were diluted to an OD_600_ of 0.2 in M9 supplemented with casamino acids. Cultures were grown for 1.5 h at 26°C. Compounds were added at indicated concentrations or the equivalent volumes of DMSO were added and the cultures switched to 37°C for another 3 h. Two hundred microliters of each culture was added to clear bottom, white 96 well plates (Costar®, Corning Inc.) and optical density and luminescence were measured using a Perkin Elmer Victor X3 plate reader. Two technical replicates were averaged for each sample per independent experiment.

### YopM translocation assay

Measuring translocation of the YopM-β-lactamase (YopM-Bla) reporter effector protein was carried out as previously described (Duncan et al., 2014). A total of 6 × 10^3^ CHO-K1 cells were plated in each well of a 384-well plate (Corning^TM^ Falcon^TM^) in 50 μl of F-12K medium plus 10% FBS and 1% glutamine, and incubated overnight. The following day, indicated concentrations of compounds, or the equivalent volumes of DMSO, were added to 26°C-grown low calcium medium cultures of *Y. pseudotuberculosis* expressing YopM-Bla and treated cultures were shifted to 37°C for 3 h. Immediately prior to infection of CHO-K1 cells, the F-12K media was removed and replaced with RPMI plus 10% FBS and compounds or DMSO were added. *Y. pseudotuberculosis* was added to the CHO-K1 plate at an MOI of 7. Five minutes after this transfer, the plate was centrifuged at 290 × g for 5 min to initiate bacterium-host cell contact and incubated for 1 h at 37°C and 5% CO_2_. Thirty minutes prior to the end of the infection, CCF2-AM (Invitrogen) was added to each well, and the plate covered in foil and incubated at room temperature. At the end of the infection, the medium was aspirated and fresh 4% paraformaldehyde added to each well for 20 min to fix the cells. The paraformaldehyde was then aspirated and the DNA dye DRAQ5 (Cell Signaling Technology) in PBS was added to each well. The monolayers were incubated at room temperature for 10 min, washed once with PBS, and visualized using an ImageXpressMICRO automated microscope and MetaXpress analysis software (Molecular Devices). The number of YopM-Bla-positive cells was calculated by dividing the number of blue (CCF2-cleaved) cells by the number of green (total CCF2) cells. Three technical replicates were averaged for each sample per independent experiment.

### Growth curves

Overnight cultures of *Y. pseudotuberculosis* were diluted to an OD_600_ of 0.1 in 2xYT and 200 μl was added to each well of a 96-well plate (Corning^TM^ Falcon^TM^). Compounds were added at 50 μM and kanamycin at 50 μg/ml, plates incubated at 26°C, and the OD_600_ of the cultures measured every hour for 13 h using a VersaMax Tunable Microplate Reader (Molecular Devices). The 96-well plates were intermittently shaken throughout the experiment. One technical replicate was used for each sample per independent experiment.

### Motility assay

Following overnight growth, 1 μl of *Y. pseudotuberculosis* overnight culture was spotted onto motility medium containing either 1% tryptone/0.25% agar or 1% tryptone/0.25% agar supplemented with 5 mM EGTA and 20 mM MgCl_2_ in six-well plates. Each well contained either 0.3% DMSO or 50 μM INP0007 or INP0010. The plates were incubated at 26°C for 24 h before the diameter of swimming motility was measured. One technical replicate was used for each sample per independent experiment.

## Results

### T3SS inhibitor training set for assay pipeline

**Figure 1:**
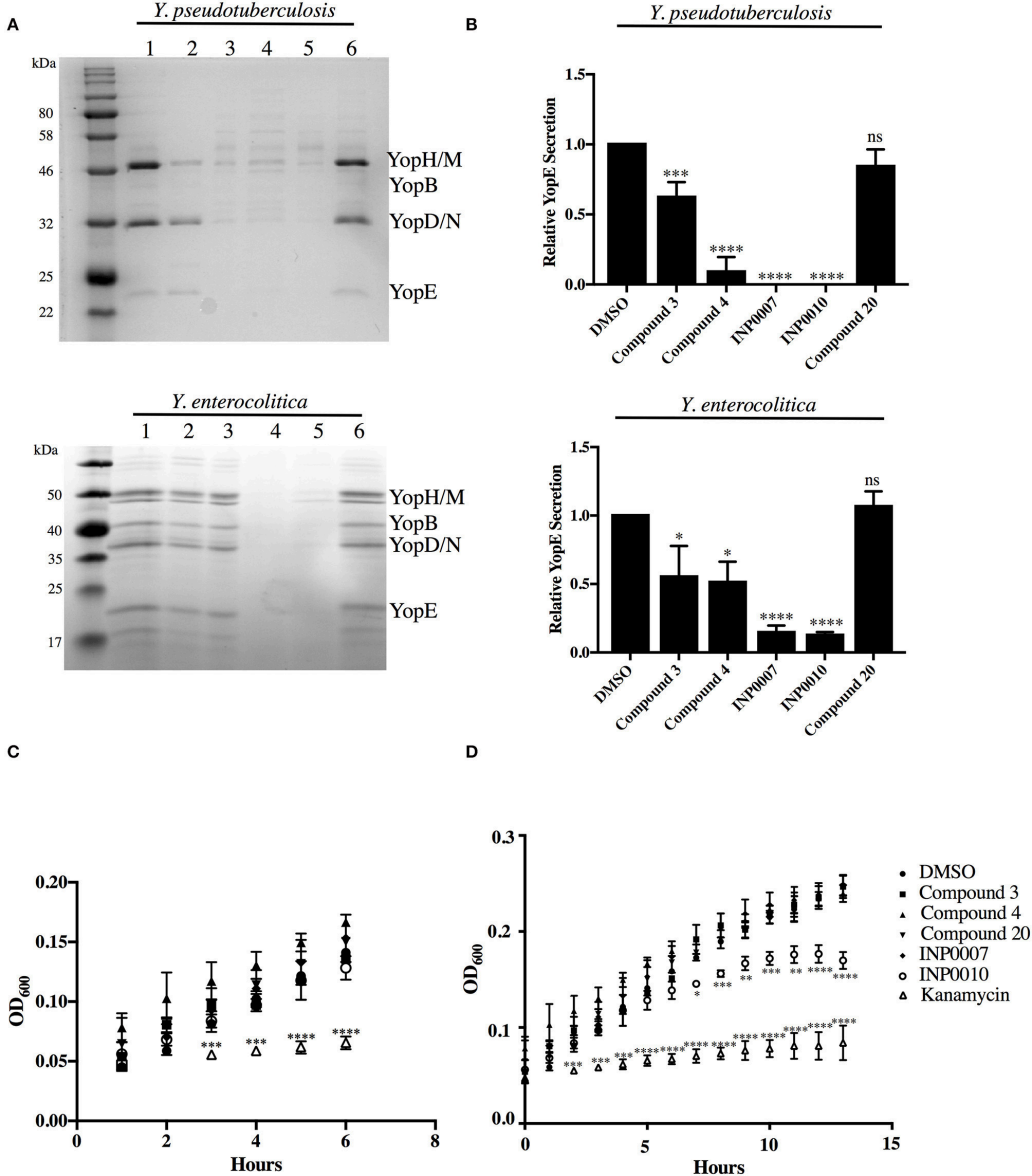
Efficiency of T3SS effector protein secretion and bacterial growth in the presence of T3SS inhibitors. **(A,B)** The relative efficiency of effector protein secretion into the culture supernatant was analyzed following bacterial growth for 2 h under T3SS-inducing conditions in the presence of either 50 μM compound or equivalent volume of DMSO. **(A)** The secretome of *Y. pseudotuberculosis* IP2666 and *Y. enterocolitica* 8081 was precipitated with trichloroacetic acid, separated by SDS-PAGE, and visualized by staining with Coomassie blue. Samples were normalized to culture optical density. (1) DMSO, (2) Compound 3, (3) Compound 4, (4) INP0007, (5) INP0010, (6) Compound 20. **(B)** Quantification of the YopE protein band by densitometry relative to the DMSO control. The average of 3 (*Y. pseudotuberculosis*) or 4 (*Y. enterocolitica*) biological replicates ± standard deviation is shown. **(C,D)** *Y. pseudotuberculosis* IP2666 growth at 26°C in the presence of 50 μM compound or DMSO was tracked by measuring optical density. The average of three biological replicates ± standard deviation is shown and statistical significance is represented comparing compounds relative to the DMSO control. *P < 0.03; **P < 0.004; ^****^P < 0.0001; ^***^P < 0.0007 (one way ANOVA with Dunnett's *post-hoc* test).

We selected a training set of commercially-available compounds to validate our assay pipeline (Table 4). All compounds selected met two strict requirements: demonstrated inhibitory activity on the *Yersinia* T3SS and absence of bactericidal activity within 6 h to accommodate the timeframe of the pipeline assays (Figures 1A–C).

**Table 4:** Compounds used in this study and their reported activity.

**Compound name**	**Chemical structure**	**Shown to inhibit:**	**References**
Compound 3 (CAS: 443329-02-0)	[-2pt] 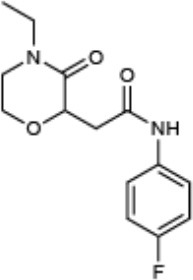	Inhibition of ExoS-Bla and YopE-Bla secretion in *Pseudomonas* and *Yersinia*, respectively	Aiello et al., 2010
Compound 4 (CAS: 138323-28-1)	[-4pt] 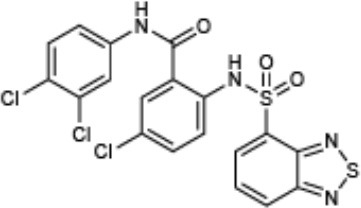	Inhibition of T3SS gene expression and effector secretion in *Yersinia*	Kauppi et al., 2003
INP0007 (CAS: 300668-15-9)	[-4pt] 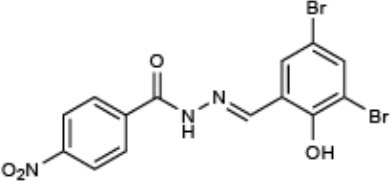	Inhibition of T3SS gene expression, effector secretion, and motility in *Yersinia* Inhibition of T3SS (SPI1) gene expression, secretion, and translocation in *Salmonella*	Kauppi et al., 2003 Nordfelth et al., 2005 Negrea et al., 2007
INP0010 (CAS: 68639-26-9)	[-4pt] 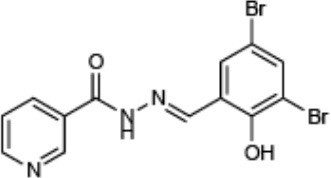	Inhibition of T3SS gene expression and effector secretion in *Yersinia* Inhibition of T3SS gene expression, secretion, and invasion in *Salmonella* Inhibition of virulence genes, including the T3SS in *E. coli*	Nordfelth et al., 2005 Negrea et al., 2007 Tree et al., 2009
Compound 20 (CAS: 489402-27-9)	[-4pt] 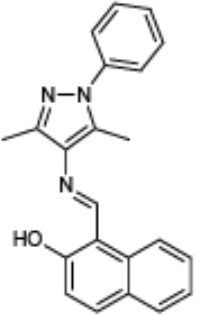	Inhibition of T3SS dependent effector translocation in *Yersinia*	Harmon et al., 2010

Compound 4 (C4), a haloid-containing sulfonamidobenzamide, was identified through a luciferase-based T3SS gene promoter fusion screen as a potent inhibitor of T3SS gene expression (Kauppi et al., 2003). We chose this compound because it was shown, albeit modestly, to inhibit expression of the LcrF master regulator of the T3SS in *Yersinia* (Kauppi et al., 2003) and we therefore expected that it would have a broad impact on all stages of type III secretion downstream of T3SS expression in our assay pipeline. From the same high throughput screening strategy that identified C4, two members of the salicylidene acylhydrazide class of inhibitors, INP0007 (Kauppi et al., 2003) and INP0010 (Nordfelth et al., 2005), were identified. Despite a large number of studies, the mechanism of action of these compounds remains unclear and there is evidence of off target effects on global virulence gene expression by at least some salicylidene acylhydrazides (Tree et al., 2009). In fact, INP0010 decreased *Yersinia* growth starting 7 h after initiation of treatment (Figure 1D), consistent with previous reports (Veenendaal et al., 2009). Compound 3 (C3), a malic diamide, was shown to inhibit ExoS secretion in *P. aeruginosa* and YopE secretion in *Yersinia pestis* (Aiello et al., 2010). This compound is structurally related to another class of T3SS inhibitors, the phenoxyacetamides, which were proposed to target the PscF needle subunit in *Pseudomonas* (Bowlin et al., 2014). Based on this structural relatedness, C3 was expected to disrupt T3SS needle assembly without impacting T3SS gene expression. Compound 20 (C20) was identified in a T3SS effector protein-β-lactamase reporter translocation screen, but had no significant effect on the ability of the bacteria to secrete T3SS effectors in the absence of host cells, pointing to the possibility that this compound specifically blocks the bacteria–host cell interaction (Harmon et al., 2010). Therefore, C20 was expected to inhibit the translocation of effector proteins into host cells but not impact T3SS gene expression and assembly.

An advantage of using Ysc-expressing *Yersinia* for our assay pipeline is that removing calcium from the culture medium enables T3SS effector proteins to be secreted into the supernatant in the absence host cells (Yother and Goguen, 1985; Perry et al., 1986; Straley and Bowmer, 1986; Sample et al., 1987; Forsberg and Wolf-Watz, 1988; Mehigh et al., 1989), providing a useful method of monitoring efficiency of T3SS inhibition in order to validate use of a given inhibitor in our assay pipeline. As expected, all test compounds except C20 exhibited significant inhibition of effector Yop secretion (Figures 1A,B). Importantly, this secretion assay was used routinely to ensure the efficacy of each batch of compound purchased and after 1 month in storage. Therefore, even when a compound had no effect in a particular assay, we could be confident that it was still active as a T3SS inhibitor.

### Assessment of T3SS gene expression

**Figure 2:**
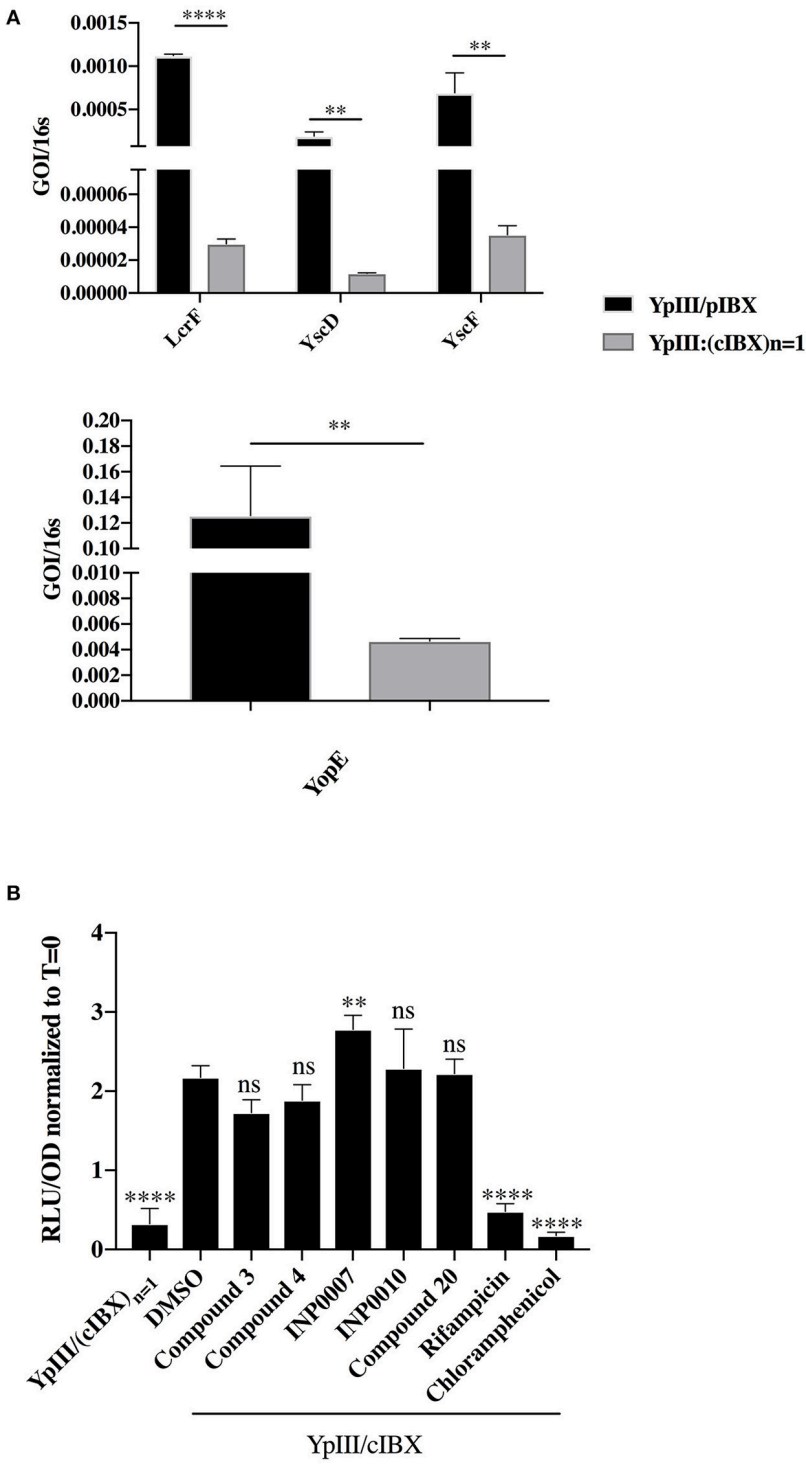
Correlation between pYV copy number and T3SS gene expression. **(A)** *Y. pseudotuberculosis* encoding luciferase genes on pYV (YPIII/pIBX) or a strain in which pYV-encoded T3SS and luciferase genes were incorporated into the chromosome in single copy [YPIII/(cIBX)_n = 1_] were grown for 3 h under T3SS-inducing conditions in 50 μM compound or equivalent volume of DMSO and T3SS gene expression evaluated by qPCR. GOI, gene of interest. Expression levels were normalized to 16s rRNA and then the fold change compared to DMSO calculated [(GOI^compound^/16s^compound^)/(GOI^DMSO^/16s^DMSO^)]. The average of four biological replicates ± standard deviation is shown. ****P < 0.0001, **P < 0.008 (Student *T*-test). **(B)** *Y. pseudotuberculosis* YpIII/pIBX and YPIII/(cIBX)_n = 1_ were grown under T3SS-inducing conditions for 3 h in 50 μM compound or equivalent volume of DMSO and luminescence measured as a readout of pYV gene copy number. The average of four biological replicates ± standard deviation is shown and statistical significance is represented comparing compounds relative to the DMSO control. ****P < 0.0001; **P = 0.008 (one way ANOVA with Dunnett's *post-hoc* test).

We reasoned that inhibition of the pYV copy number upregulation that occurs during normal induction of type III secretion in *Yersinia* may impact T3SS gene expression, and therefore affect overall T3SS activity. To test this, we employed a *Y. pseudotuberculosis* strain with a luciferase reporter gene cluster integrated onto the pYV virulence plasmid (YpIII/pIBX) (Fahlgren et al., 2014). As a negative control, we used a strain in which a replication deficient copy of the virulence plasmid as well as the luciferase gene cluster was integrated into the chromosome (referred to as YpIII/pIBX_N = 1_) (Wang et al., 2016). In this YpIII/pIBX_N = 1_ strain, the ratio of T3SS and luciferase reporter genes vs. chromosomal genes remains constant even upon induction of type III secretion (Wang et al., 2016). As expected, expression of *lcrF*, the effector protein *yopE*, the integral IM ring protein *yscD*, and the needle subunit *yscF* were significantly decreased in the YpIII/pIBX_N = 1_ strain compared to the YpIII/pIBX strain (Figure 2A). While these data demonstrate that inhibition of pYV gene copy number could impact pYV gene expression, four of the five compounds in our training set did not significantly affect virulence plasmid copy number as measured by luciferase activity, while INP0007 significantly increased copy number (*p* = 0.0054; Figure 2B) for reasons that are unclear but that may reflect off target effects. In contrast, luciferase expression could be inhibited by blocking general transcription or translation using rifampicin or chloramphenicol (Figure 2B). Therefore, while inhibiting pYV copy number in *Yersinia* causes a decrease in T3SS activity, we conclude that none of the compounds in our training set act by inhibiting upregulation of pYV copy number.

**Figure 3:**
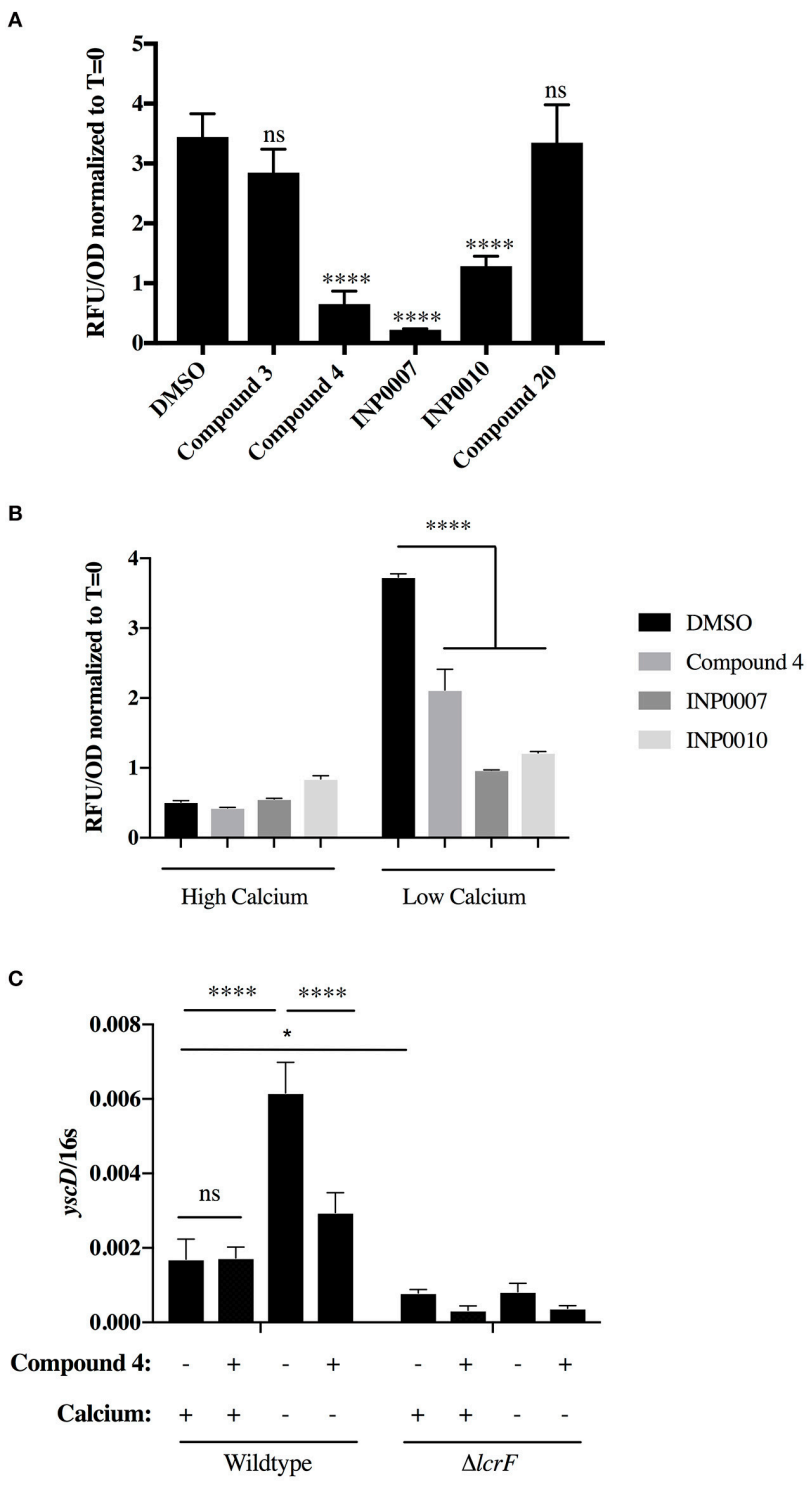
Analysis of *yopH*-mCherry fluorescence to assess T3SS gene expression. **(A,B)** *Y. pseudotuberculosis* p*yopH* FLAG mCherry was grown under T3SS-inducing (**A,B**, low calcium) or non-inducing (**B**, high calcium) conditions and relative mCherry fluorescence measured at 3 h after addition of 50 μM compound or equivalent volume of DMSO. The average of three biological replicates ± standard deviation is shown and statistical significance is represented comparing compounds relative to the DMSO control. ****P < 0.0001 (one way ANOVA with Dunnett's *post-hoc* test). **(C)** *Y. pseudotuberculosis* wildtype or Δ*lcrF* were grown in low or high calcium media for 3 h in 50 μM Compound 4 or equivalent volume of DMSO and *yscD* mRNA levels measured by qPCR. Expression levels were normalized to 16s rRNA. Statistical significance is represented comparing the indicated pairs of conditions. ****P < 0.0001, *P < 0.002 (Student *t*-test).

**Figure 4:**
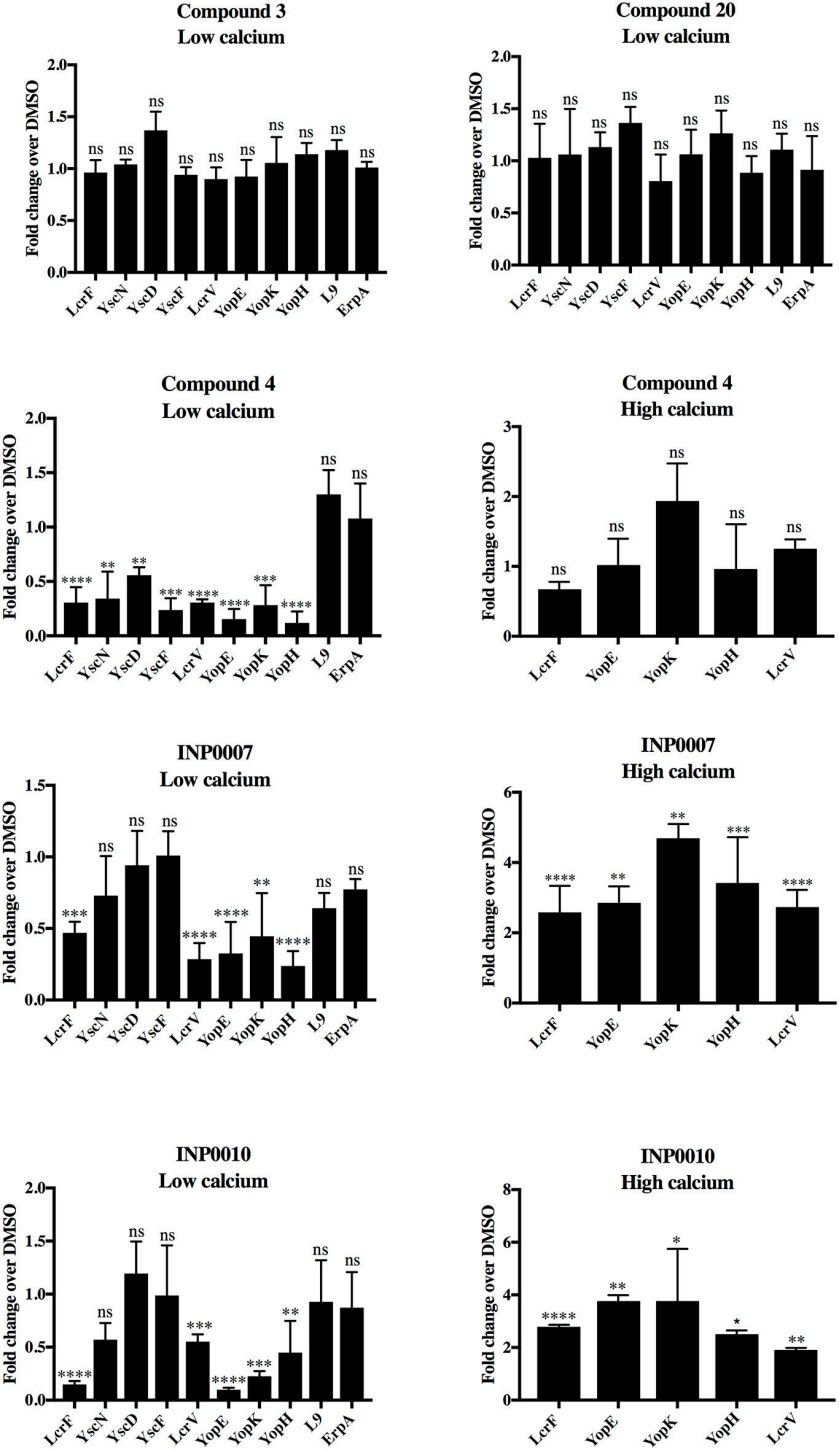
T3SS gene mRNA levels under low and high calcium conditions. Wildtype *Y. pseudotuberculosis* was grown under high or low calcium conditions in the presence of 50 μM compound or equivalent volume of DMSO and mRNA levels of T3SS (*lcrF, yscN, yscD, yscF, lcrV, yopE, yopK, yopH*) and non-T3SS genes (L9, *erpA*) assessed by qPCR. The average of four biological replicates ± standard deviation is shown and statistical significance is represented comparing compounds relative to the DMSO control for each gene. ****P < 0.0001, ***P < 0.0005, **P < 0.003; *P < 0.022 (one way ANOVA with Dunnett's *post-hoc* test). ns, not significant.

In order to determine if the training set compounds negatively impacted T3SS gene expression, we used a *Yersinia* reporter strain containing an unstable YopH-mCherry-AAV transcriptional reporter (Andersen et al., 1998). C4, INP0007, and INP0010 inhibited YopH-mCherry-AAV expression when T3SS activity was induced by incubation in low calcium medium at 37°C (Figure 3A). In order to validate and extend these results, we measured transcript levels of T3SS and non-T3SS genes by qPCR. Consistent with the YopH-mCherry-AAV data, C3 and C20 had no significant impact on gene expression (Figure 4). However, C4 inhibited expression of all T3SS genes tested, but did not impact expression of two non-T3SS-associated genes: the iron sulfur cluster loading protein *erpA* and the small ribosomal subunit L9. Interestingly, INP0007 and INP0010 significantly inhibited transcript levels of *lcrF*, the needle tip subunit *lcrV*, and effector proteins *yopH, yopE*, and *yopK*, but did not inhibit expression of the ATPase *yscN, yscF*, and *yscD* under these conditions (Figure 4). To test whether inhibition of secretion via the positive feedback loop indirectly inhibits T3SS gene expression, we measured YopH-mCherry fluorescence and *yscD* mRNA levels via qPCR during growth at 37°C in high calcium (Figures 3B,C). Under these conditions, *Yersinia* can assemble the T3SS (Diepold et al., 2010), but no secretion of Yop effectors occurs (Forsberg and Wolf-Watz, 1988); therefore, the positive feedback loop is not active in high calcium. While overall expression levels of YscD and YopH are lower in high calcium compared to low calcium conditions, C4, INP0007, and INP0010 did not significantly decrease YopH-mCherry-AAV fluorescence in high calcium conditions (Figure 3B), suggesting an indirect effect of these compounds on T3SS gene expression through inhibition of secretion in low calcium conditions. Likewise, *yscD* levels were not decreased by C4 in high calcium (Figure 3C). Importantly, *yscD* expression was significantly lower in the Δ*lcrF* mutant compared to WT under high calcium conditions in the absence of compound. These data indicate that C4 is not likely to target LcrF activity, as *yscD* is predicted to be under LcrF control (Schwiesow et al., 2015).

### Assessment of T3SS assembly

**Figure 5:**
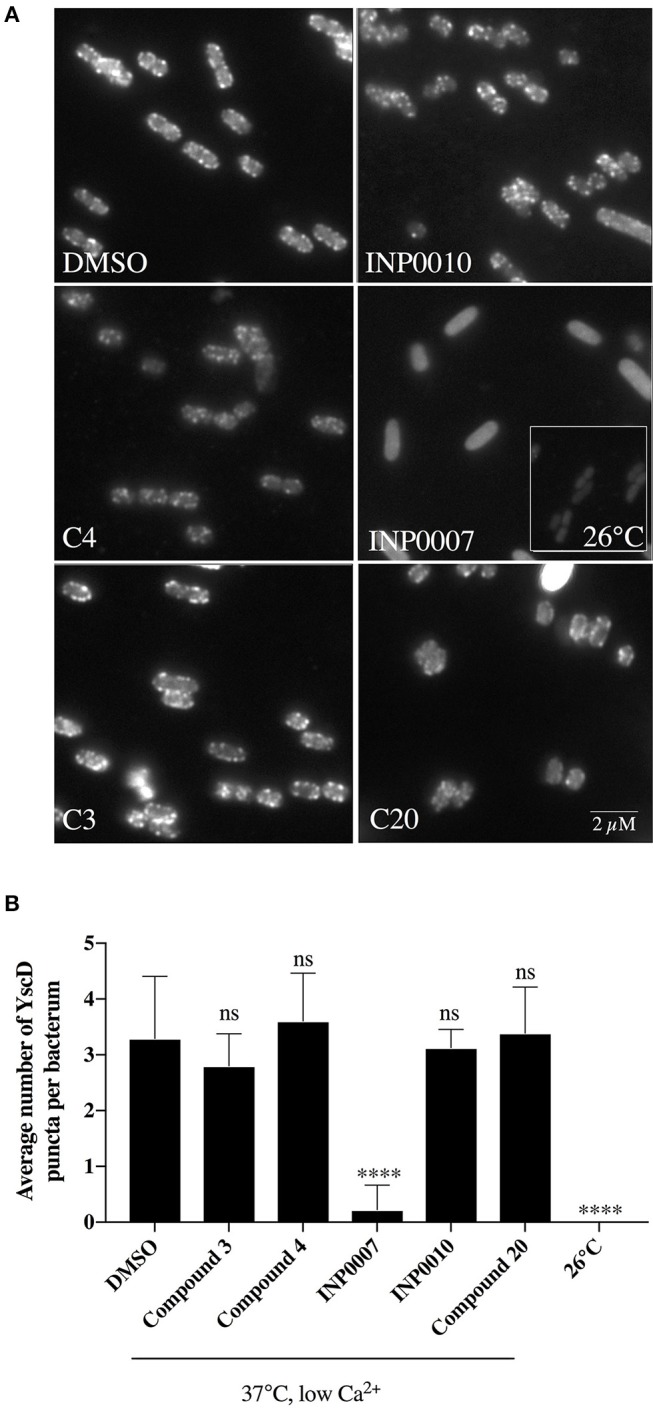
YscD-EGFP puncta formation in *Y. enterocolitica. Y. enterocolitica* pYV40-EGFP-*yscD* was grown under T3SS-inducing conditions for 3 h in the presence of 50 μM compound or equivalent volume of DMSO and fluorescent foci quantified using IMARIS software. Representative images **(A)** and the average number of foci ± standard deviation per cell **(B)** are shown from three biological replicates, with *N* > 2,000 cells quantified per condition. Statistical significance is represented comparing compounds relative to the DMSO control. ****P < 0.008 (one way ANOVA with Dunnett's *post-hoc* test).

**Figure 6:**
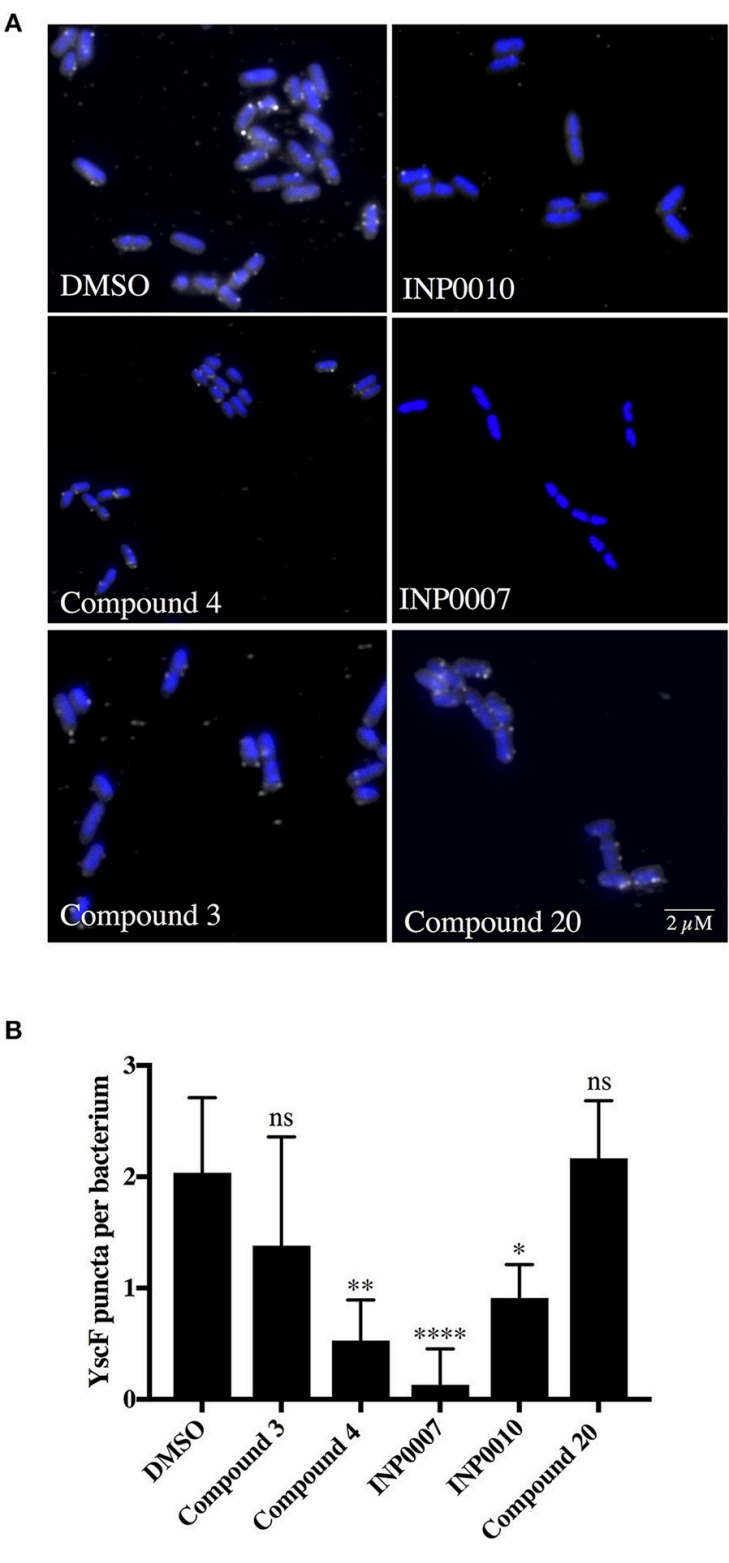
YscF puncta visualization in *Y. pseudotuberculosis* using immunofluorescence. Wildtype *Y. pseudotuberculosis* was grown under T3SS-inducing conditions for 3 h in 50 μM compound or equivalent volume of DMSO and an anti-YscF antibody used to visualize T3SS needles. **(A)** Representative confocal microscopy images and **(B)** the average number of YscF puncta per bacterium from three biological replicates ± the standard deviation are shown, with *N* > 1,500 bacteria quantified per condition. Statistical significance is represented comparing compounds relative to the DMSO control. ****P < 0.0001, **P < 0.002, *P < 0.04 (one way ANOVA with Dunnett's *post-hoc* test).

In order to monitor T3SS assembly, we used fluorescence microscopy to quantify YscD localization in *Y. enterocolitica* and YscF puncta formation in *Y. pseudotuberculosis* as a proxy for T3SS basal body and T3SS needle assembly, respectively, as previously described (Davis and Mecsas, 2007; Diepold et al., 2010; Morgan et al., 2017). Only INP0007 significantly impacted YscD-EGFP puncta formation (Figure 5). Importantly, the level of diffuse fluorescence in INP0007-treated *Yersinia* was greater than that of the non-T3SS-inducing condition (26°C-grown *Yersinia*; Figure 5A, inset), indicating that while YscD is expressed at 37°C in the presence of INP0007, it is not assembled into basal bodies in the presence of this compound. In contrast, while C3 and C20 had no significant effect on YscF puncta formation as measured with an anti-YscF antibody, C4, INP0007, and INP0010 significantly inhibited needle assembly (Figure 6).

**Figure 7:**
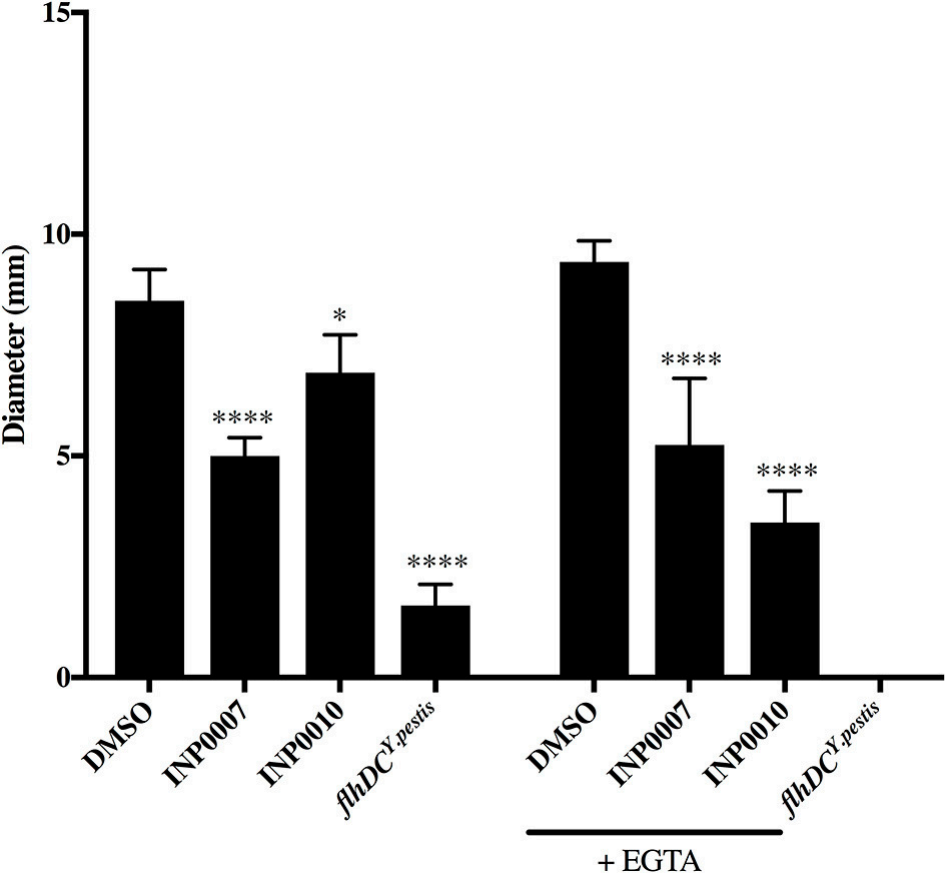
INP0007 and INP0010 inhibit *Y. pseudotuberculosis* motility. *Y. pseudotuberculosis* wildtype or the non-motile *flhDC*^*Y*.*pestis*^ mutant were spotted onto motility agar containing the 50 μM compounds or the equivalent volume of DMSO and allowed to grow for ~24 h. Average swimming diameter of the colony was measured from three biological replicates ± standard deviation and statistical significance is represented comparing compounds relative to the DMSO control. ****P < 0.0001, *P < 0.03 (one way ANOVA with Dunnett's *post-hoc* test).

The bacterial flagellar apparatus is composed of a basal body that is structurally related to the injectisome T3SS basal body and mediates secretion of the flagellar hook and filament proteins (Macnab, 2004). Therefore, it is possible that compounds with the ability to inhibit injectisome T3SS basal body assembly may inhibit flagellar assembly and therefore flagellar motility. INP0007 has been shown to inhibit motility in *Yersinia* (Kauppi et al., 2003), but neither INP0007 nor INP0010 inhibited motility in *Salmonella* (Negrea et al., 2007). In our standard *Yersinia* motility agar (Morgan et al., 2017), INP0007 significantly decreased flagellar motility in *Y. pseudotuberculosis*, while INP0010 decreased motility weakly albeit significantly (Figure 7). Addition of the chelating agent EGTA in the motility agar, as used in the Kauppi et al study (Kauppi et al., 2003), led to an even greater inhibitory effect on motility by INP0010 (Figure 7). The *Y. pseudotuberculosis flhDC*^*Ypestis*^ non-motile strain served as the negative control for the motility assay. Taken together, these data indicate that INP0007 and INP0010 impact both the flagellar and injectisome T3SS.

### Assessment of effector protein secretion *in vitro*

**Figure 8:**
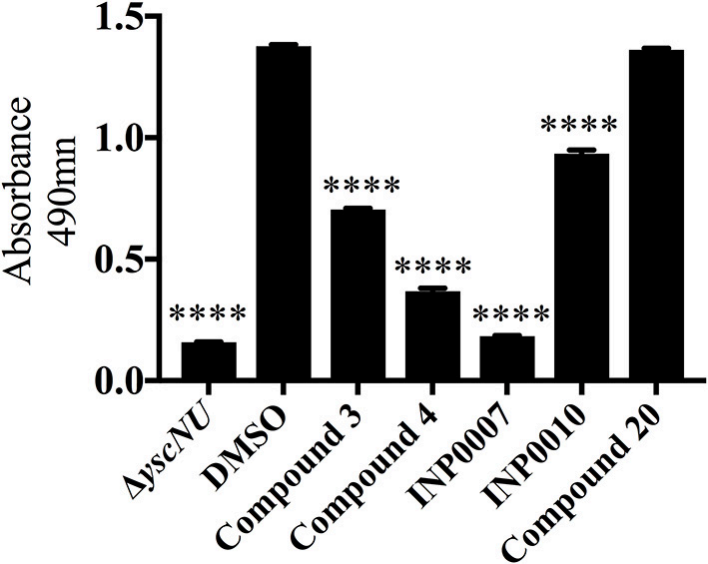
Secretion of YopM-Bla into the culture supernatant. *Y. pseudotuberculosis* Δyop6 expressing the Yop reporter YopM-Bla were grown under T3SS-inducing *in vitro* conditions for 2 h in the presence of 50 μM compound or equivalent volumes of DMSO and cleavage of the β-lactamase substrate nitrocefin used to measure the relative quantity of secreted YopM-Bla. The average of three independent experiments ± standard deviation are shown and statistical significance is represented comparing compounds relative to the DMSO control. ****P < 0.0001 (one way ANOVA with Dunnett's *post-hoc* test).

While the Yop *in vitro* secretion assay shown in Figure 1 is critical for assessing the impact of a compound on overall Yop secretion, the method used (Coomassie staining of SDS-PAGE separated proteins precipitated from the culture supernatant) is not amenable to a high throughput format. In order to assess Yop secretion *in vitro* in microtiter plates, *Y. pseudotuberculosis* expressing a YopM-β-lactamase (YopM-Bla) reporter was used in conjunction with the chromogenic β-lactamase substrate nitrocefin (O'Callaghan et al., 1972; Lee et al., 2007; Green et al., 2016). Consistent with the block in Yop secretion observed in Figure 1, C3, C4, INP0007, and INP0010 significantly blocked YopM-Bla secretion into the culture supernatant while C20 did not (Figure 8). Surprisingly, INP0010 blocked YopM-Bla secretion by only 32% as measured using nitrocefin (Figure 8), yet appeared to have a much more significant effect on native YopM secretion as measured using Coomassie staining (Figure 1). The reason for this discrepancy is unclear; however, we note that INP0010 has cytotoxic effects on bacteria and eukaryotic cells (see below) and is likely to have the greatest off target effects of the compounds in our training set.

### Assessment of effector protein translocation

**Figure 9:**
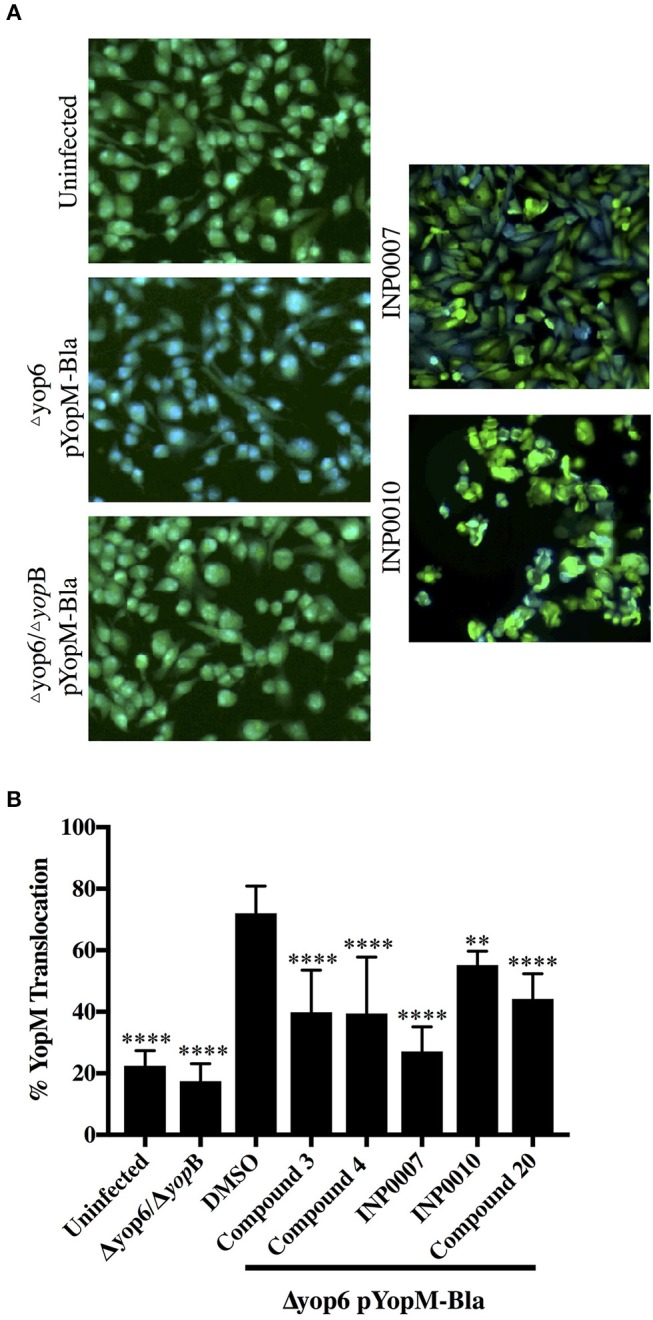
Translocation of YopM-Bla into mammalian cells in the presence of T3SS inhibitors. **(A)** Representative fluorescent micrographs of CCF2-AM loaded CHO K1 cells in the absence of bacteria (uninfected) or infected for 2 h with *Y. pseudotuberculosis* lacking YopHEMOJT but expressing the Yop reporter YopM-Bla (Δyop6 pYopM-Bla) at MOI 7 in the presence of 50 μM compound or equivalent volume of DMSO. A T3SS-defective mutant lacking the pore-forming protein YopB was used as a negative control (Δyop6/Δ*yopB* pYopM-Bla). **(B)** CCF2 green (uncleaved) and blue (cleaved) fluorescence was measured and the average from three biological replicates ± standard deviation is shown and statistical significance is represented relative to the DMSO control. ****P < 0.0001, **P < 0.007 (one way ANOVA with Dunnett's *post-hoc* test).

One of the most important requirements of a T3SS inhibitor is its ability to prevent translocation of effector proteins into target host cells. We employed a different type of YopM-Bla reporter assay to assess translocation of effector proteins into CHO K1 cells loaded with CCF2, a fluorescent β-lactamase substrate that can enter eukaryotic cells (Dewoody et al., 2011). While C3, C4, C20, INP0007, and INP0010 significantly blocked YopM-Bla translocation at 50 μM, INP0010 appeared to be cytotoxic at this concentration (Figure 9).

## Discussion

A methodical approach to primary characterization of inhibitors for the T3SS has been lacking in the field. Here, we describe a pipeline of miniaturized assays, using enteropathogenic *Yersinia* as the workhorse organism, that enable rapid, initial characterization of the stage of T3SS expression, assembly, or function targeted by compounds with T3SS inhibitory activity. Furthermore, we used a training set of compounds with previously-identified T3SS inhibitory activity to test the utility of this pipeline (Table 5). INP0007 blocked T3SS basal body and needle assembly and therefore inhibited Yop secretion and translocation. C4 and INP0010 did not prevent basal body assembly but blocked needle assembly, secretion, and translocation. However, INP0010 exhibited cytotoxicity, complicating interpretation of some assay results. C3 allowed T3SS assembly but prevented Yop secretion and translocation. Lastly, C20 allowed T3SS assembly and Yop secretion *in vitro*, but blocked translocation of Yops into target host cells. These results demonstrate the ability of our assay pipeline both to validate T3SS inhibitors and provide testable hypotheses on their mode of action.

**Table 5:** Summary of experimental pipeline assays using our training set of T3SS inhibitors.

**Compound name**	**pYV copy number^a^**	**T3SS gene expression (high Ca^2+^)^b^**	**T3SS gene expression (low Ca^2+^)^c^**	**YscD puncta per bacterium^c^**	**YscF puncta per bacterium^c^**	**Secretion (%DMSO)^d^**	**Translocation^e^ (%)**
DMSO (control)				~3.3	~2.0	100	~72
C3	No change	No change	No change	~2.8	~1.38	51	~40
C4	No change	No change	All T3SS genes tested	~3.6	~0.5	27	~39
INP0007	Increase	No change	Subset of T3SS genes tested	~0.2	~0.13	13	~27
INP0010	No change	No change	Subset of T3SS genes tested	~3.1	~0.92	68	~55
C20	No change	No change	No change	~3.4	~2.1	99	~44

*Dark gray boxes represent statistically significant inhibitory activity at a distinct stage of T3SS deployment. Light gray boxes represent significant inhibitory activity on T3SS gene expression only under low calcium conditions, when the positive feedback loop is active and strong inhibition of secretion leads to a decrease in T3SS gene expression*.

a *Compared to DMSO control, as measured by pYV-encoded luciferase activity*.

b *Compared to DMSO control under high calcium conditions, as measured by qPCR or monitoring YopH-mCherry expression*.

c *Under low calcium conditions*.

d *Compared to DMSO control, as measured by nitrocefin color change resulting from YopM-Bla secreted into the culture supernatant under low calcium conditions*.

e *Percent of cells with β-lactamase substrate CCF2 blue fluorescence in CHO cells*.

C3 blocked the ability of the *Yersinia* T3SS to secrete T3SS effector proteins into low calcium culture supernatant and to translocate effector proteins into target host cells. Yet C3 did not block T3SS basal body and needle assembly, as determined by imaging YscD basal body and YscF needle puncta formation. C3 showed a modest inhibition of YopE secretion (28%), but notably inhibited secretion of the translocator protein YopD (Figure 1A). These data provide a possible explanation for why C3 strongly inhibited Yop translocation into host cells (40%) despite less potent inhibition of YopE secretion, as YopD is required for Yop delivery into host cells (Rosqvist et al., 1994) C3 has structural similarities with the phenoxyacetamides, which target the SctF needle subunit (Aiello et al., 2010; Bowlin et al., 2014). However, our data indicate that C3 blocks a specific activity of the assembled T3SS. Alternatively, the T3SS needle structure may be altered in the presence of C3, still allowing recognition by our anti-YscF antibody but impeding cargo egress.

In contrast to C3, C20 specifically blocked translocation of a T3SS effector protein into host cells without blocking any other stage of type III secretion, including T3SS effector protein secretion into low calcium culture. As C20 was previously shown to inhibit only translocation of T3SS effector proteins into target host cells (Harmon et al., 2010), our verification of this finding further demonstrates the robustness of our experimental pipeline. Harmon et al. hypothesized that C20 inhibited the host cell-bacterial interaction, as C20 strongly inhibited adherence of *Y. pseudotuberculosis* to HEp-2 cells. Possible modes of action of compounds that inhibit Yop translocation but not any other stage of type III secretion include interruption of bacterial adhesins, host integrin receptors, or YopBD-mediated pore formation on the host membrane.

C4 was shown to inhibit promoter activity of *lcrF*, the Ysc T3SS master regulator (Kauppi et al., 2003). However, while we observed a C4-dependent decrease in mRNA levels of all T3SS genes tested under low calcium T3SS-inducing conditions, C4 treatment did not impact T3SS gene expression under high calcium conditions when T3SSs are assembled but no Yop secretion occurs. As a Δ*lcrF* mutant had significantly less T3SS gene expression under high calcium conditions compared to wildtype *Yersinia*, this argues against C4 inhibiting LcrF activity. C4 did not alter the number of YscD puncta observed but did decrease the number of YscF puncta per cell by greater than half. These data suggest that C4 does not impact YscD assembly in the plasma membrane but disrupts overall T3SS basal body formation in such a way that needle formation is compromised or interferes with YscF secretion or assembly.

The salicylidene acylhydrazides INP0007 and INP0010 inhibited expression of *lcrF, lcrV*, and *yopKEH*, but did not decrease expression of *yscN, yscD*, or *yscF*. While INP0007 and INP0010 are related structurally, INP0010 prevents *Yersinia* growth if the bacteria are exposed longer than 6 h, while INP0007 does not affect bacterial growth even up to 13 h of exposure. Surprisingly, while INP0010 did not inhibit YscD puncta formation, INP0007 caused a 10-fold decrease in YscD puncta. Both compounds significantly inhibited YscF puncta formation, Yop secretion into low calcium culture, and Yop translocation into host cells. However, measuring Yop translocation into host cells in the presence of INP0010 is complicated by the obvious toxic effects of the compound on host cells. Therefore, utilization of the Yop translocation assay of our experimental pipeline is limited to compounds without toxic effects on host cells. However, this also shows the benefit of using microscopy to observe CCF2 green-to-blue fluorescence conversion, as it allows observation of host cell morphology. The salicylidene acylhydrazide class of compounds have been suggested to have a broad impact on expression of horizontally acquired genes (Tree et al., 2009). However, we only observe a defect in T3SS gene expression in low calcium, but not high calcium medium, for INP0007. In addition, we observe a significant defect in flagellar motility by INP0007, suggesting a broader effect of the compound either on the flagellar T3SS, ATP synthesis, or the proton motive force. In total, our results argue that INP0007 inhibits formation of the injectisome T3SS and, either through a similar mechanism or by targeting multiple pathways, also disrupts flagellar T3SS activity. Therefore, we suggest that for T3SS inhibitors that block T3SS basal body assembly, a flagellar motility assay be performed to assess their breadth of action.

Given the previously-demonstrated positive feedback exerted by active type III secretion on T3SS gene transcription (Cornelis et al., 1987), we expected that C3 would inhibit T3SS gene expression in low calcium medium. C4 and INP0007 inhibited YopE secretion by >70–80% and this correlated with the ability to affect T3SS gene expression under low calcium conditions. C3 inhibited YopE secretion < 50% and this was insufficient to impact the feedback loop on T3SS gene expression. Similarly, the T3SS inhibitor piericidin A1 inhibits YopE secretion by ~40–45% and also does not repress transcription of T3SS genes (Morgan et al., 2017). These data suggest that only compounds that potently inhibit Yop secretion will affect the T3SS gene expression feedback loop. This indicates that screening strategies based on Yop gene expression as the readout for T3SS inhibition may miss less robust compounds that could be improved by structure-activity relationship analysis.

While none of the inhibitors we tested affected pYV copy number, our data shows that prevention of pYV plasmid copy number upregulation during active type III secretion leads to dramatically reduced T3SS gene mRNA steady-state levels. Therefore, when using *Yersinia* as a model organism for assessing T3SS inhibitor mechanism of action, it is important to consider pYV copy number. Furthermore, as chloramphenicol and rifampicin significantly inhibited luminescence as a readout of pYV copy number, this assay also sheds light on whether a compound affects general transcription or translocation. One caveat is that compounds that act as luminescence quenchers would decrease luminescence in this assay. In this case, analyzing expression of non-T3SS genes, such as the L9 and ErpA genes shown here, via qPCR can serve as a way to test this possibility.

In summary, we propose that the experimental pipeline described here can be used to rapidly bin T3SS inhibitors into categories depending on the stage of type III secretion they inhibit, providing testable hypotheses on mode of action. The commercially available training set shown here can be used to establish these assays in other labs and perhaps enable a more standardized approach to T3SS inhibitor research.

## Author contributions

JM: experimental design, performing experiments, writing paper; HL: experimental design, performing experiments; JD and JL: performing experiments; SM, RI, HW: providing reagents; VA: experimental design, writing paper.

### Conflict of interest statement

The authors declare that the research was conducted in the absence of any commercial or financial relationships that could be construed as a potential conflict of interest.

## References

[B1] Aiello D. Williams J. D. Majgier-Baranowska H. Patel I. Peet N. P. Huang J.. (2010). Discovery and characterization of inhibitors of *Pseudomonas aeruginosa* type III secretion. Antimicrob. Agents Chemother. 54, 1988–1999. 10.1128/AAC.01598-09 20176902 PMC2863679

[B2] Anantharajah A. Buyck J. M. Sundin C. Tulkens P. M. Mingeot-Leclercq M. P. Van Bambeke F. (2017). Salicylidene acylhydrazides and hydroxyquinolines act as inhibitors of type three secretion systems in *Pseudomonas aeruginosa* by distinct mechanisms. Antimicrob. Agents Chemother. 61:e02566-16. 10.1128/AAC.02566-16 28396545 PMC5444141

[B3] Andersen J. B. Sternberg C. Poulsen L. K. Bjorn S. P. Givskov M. Molin S. (1998). New unstable variants of green fluorescent protein for studies of transient gene expression in bacteria. Appl. Environ. Microbiol. 64, 2240–2246. 9603842 10.1128/aem.64.6.2240-2246.1998 PMC106306

[B4] Auerbuch V. Golenbock D. T. Isberg R. R. (2009). Innate immune recognition of *Yersinia pseudotuberculosis* type III secretion. PLoS Pathog. 5:e1000686. 10.1371/journal.ppat.1000686 19997504 PMC2779593

[B5] Beckham K. S. Roe A. J. (2014). From screen to target: Insights and approaches for the development of anti-virulence compounds. Front. Cell. Infect. Microbiol. 4:139. 10.3389/fcimb.2014.00139 25325019 PMC4179734

[B6] Bergeron J. Worrall L. Sgourakis N. DiMaio F. Pfuetzner R. Felise H.. (2013). A refined model of the prototypical *Salmonella* spi-1 t3ss basal body reveals the molecular basis for its assembly. PLoS Pathog. 9:e1003307. 10.1371/journal.ppat.1003307 23633951 PMC3635987

[B7] Berube B. J. Murphy K. R. Torhan M. C. Bowlin N. O. Williams J. D. Bowlin T. L.. (2017). Impact of type III secretion effectors and of phenoxyacetamide inhibitors of type III secretion on abscess formation in a mouse model of *Pseudomonas aeruginosa* infection. Antimicrob. Agents Chemother. 61:e01202–17. 10.1128/AAC.01202-17 28807906 PMC5655109

[B8] Bliska J. B. Guan K. L. Dixon J. E. Falkow S. (1991). Tyrosine phosphate hydrolysis of host proteins by an essential *Yersinia* virulence determinant. Proc. Natl. Acad. Sci. USA. 88, 1187–1191. 1705028 10.1073/pnas.88.4.1187 PMC50982

[B9] Bowlin N. O. Williams J. D. Knoten C. A. Torhan M. C. Tashjian T. F. Li B.. (2014). Mutations in the *Pseudomonas aeruginosa* needle protein gene *pscF* confer resistance to phenoxyacetamide inhibitors of the type III secretion system. Antimicrob. Agents Chemother. 58, 2211–2220. 10.1128/AAC.02795-13 24468789 PMC4023729

[B10] Broz P. Mueller C. A. Müller S. A. Philippsen A. Sorg I. Engel A.. (2007). Function and molecular architecture of the *Yersinia* injectisome tip complex. Mol. Microbiol. 65, 1311–1320. 10.1111/j.1365-2958.2007.05871.x 17697254

[B11] Büttner D. Bonas U. (2002). Port of entry–the type III secretion translocon. Trends Microbiol. 10, 186–192. 10.1016/S0966-842X(02)02331-4 11912026

[B12] Coburn B. Sekirov I. Finlay B. (2007). Type III secretion systems and disease. Clin. Microbiol. Rev. 20, 535–549. 10.1128/CMR.00013-07 17934073 PMC2176049

[B13] Cornelis G. Vanootegem J. C. Sluiters C. (1987). Transcription of the yop regulon from *Y. enterocolitica* requires trans acting pYV and chromosomal genes. Microb Pathog. 2, 367–379. 3507556 10.1016/0882-4010(87)90078-7

[B14] Davis A. Mecsas J. (2007). Mutations in the *Yersinia pseudotuberculosis* type III secretion system needle protein, *yscF*, that specifically abrogate effector translocation into host cells. J. Bacteriol. 189, 83–97. 10.1128/JB.01396-06 17071752 PMC1797200

[B15] Deng W. Marshall N. C. Rowland J. L. McCoy J. M. Worrall L. J. Santos A. S. (2017). Assembly, structure, function and regulation of type III secretion systems. Nat. Rev. Microbiol. 15, 323–337. 10.1038/nrmicro.2017.20 28392566

[B16] Dewoody R. Merritt P. M. Houppert A. S. Marketon M. M. (2011). YopK regulates the *Yersinia pestis* type III secretion system from within host cells. Mol. Microbiol. 79, 1445–1461. 10.1111/j.1365-2958.2011.07534.x 21205017 PMC3210821

[B17] Diepold A. Amstutz M. Abel S. Sorg I. Jenal U. Cornelis G. R. (2010). Deciphering the assembly of the yersinia type III secretion injectisome. EMBO J. 29, 1928–1940. 10.1038/emboj.2010.84 20453832 PMC2885934

[B18] Duncan M. C. Linington R. G. Auerbuch V. (2012). Chemical inhibitors of the type three secretion system: disarming bacterial pathogens. Antimicrob. Agents Chemother.. 56(11):5433–5441. 10.1128/AAC.00975-12 22850518 PMC3486574

[B19] Duncan M. C. Wong W. R. Dupzyk A. J. Bray W. M. Linington R. G. Auerbuch V. (2014). An nF-κβ-based high-throughput screen identifies piericidins as inhibitors of the *Yersinia pseudotuberculosis* type III secretion system. Antimicrob. Agents Chemother. 58, 1118–1126. 10.1128/AAC.02025-13 24295981 PMC3910828

[B20] Fahlgren A. Avican K. Westermark L. Nordfelth R. Fällman M. (2014). Colonization of cecum is important for development of persistent infection by *Yersinia pseudotuberculosis*. Infect. Immun. 82, 3471–3482. 10.1128/IAI.01793-14 24891107 PMC4136198

[B21] Forsberg A. Wolf-Watz H. (1988). The virulence protein yop5 of *Yersinia pseudotuberculosis* is regulated at transcriptional level by plasmid-plb1 -encoded trans-acting elements controlled by temperature and calcium. Mol. Microbiol. 2, 121–133. 10.1111/j.1365-2958.1988.tb00013.x 28776790

[B22] Francis M. S. Wolf-Watz H. Forsberg A. (2002). Regulation of type III secretion systems. Curr. Opin. Microbiol. 5, 166–172. 10.1016/S1369-5274(02)00301-6 11934613

[B23] Green E. R. Clark S. Crimmins G. T. Mack M. Kumamoto C. A. Mecsas J. (2016). Fis is essential for *Yersinia pseudotuberculosis* virulence and protects against reactive oxygen species produced by phagocytic cells during infection. PLoS Pathog. 12:e1005898. 10.1371/journal.ppat.1005898 27689357 PMC5045184

[B24] Harmon D. E. Davis A. J. Castillo C. Mecsas J. (2010). Identification and characterization of small-molecule inhibitors of yop translocation in *Yersinia pseudotuberculosis*. Antimicrob. Agents Chemother. 54, 3241–3254. 10.1128/AAC.00364-10 20498321 PMC2916352

[B25] Heroven A. K. Böhme K. Dersch P. (2012). The Csr/Rsm system of *Yersinia* and related pathogens: a post-transcriptional strategy for managing virulence. RNA Biol. 9, 379–391. 10.4161/rna.19333 22336760

[B26] Horton R. M. Cai Z. L. Ho S. N. Pease L. R. (1990). Gene splicing by overlap extension: tailor-made genes using the polymerase chain reaction. BioTechniques 8, 528–535. 2357375

[B27] Karzai A. W. Roche E. D. Sauer R. T. (2000). The ssrA-smpB system for protein tagging, directed degradation and ribosome rescue. Nat. Struct. Biol. 7, 449–455. 10.1038/75843 10881189

[B28] Kauppi A. M. Nordfelth R. Uvell H. Wolf-Watz H. Elofsson M. (2003). Targeting bacterial virulence: inhibitors of type III secretion in *Yersinia*. Chem. Biol. 10, 241–249. 10.1016/S1074-5521(03)00046-2 12670538

[B29] Lee V. T. Pukatzki S. Sato H. Kikawada E. Kazimirova A. A. Huang J.. (2007). Pseudolipasin a is a specific inhibitor for phospholipase A2 activity of *Pseudomonas aeruginosa* cytotoxin exoU. Infect. Immun. 75, 1089–1098. 10.1128/IAI.01184-06 17178785 PMC1828555

[B30] Macnab R. M. (2004). Type III flagellar protein export and flagellar assembly. Biochim. Biophys. Acta. 1694, 207–217. 10.1016/j.bbamcr.2004.04.005 15546667

[B31] Marsden A. E. King J. M. Spies M. A. Kim O. K. Yahr T. L. (2015). Inhibition of Pseudomonas aeruginosa ExsA DNA binding activity by H-hydroxybenzimidazoles. Antimicro. Agents Chemother. 60, 766–776. 10.1128/AAC.02242-15 26574012 PMC4750669

[B32] Marshall N. C. Finlay B. B. (2014). Targeting the type III secretion system to treat bacterial infections. Expert Opin. Ther. Targets 18, 137–152. 10.1517/14728222.2014.855199 24295327

[B33] Mehigh R. J. Sample A. K. Brubaker R. R. (1989). Expression of the low calcium response in *Yersinia pestis*. Microb. Pathog. 6, 203–217. 10.1016/0882-4010(89)90070-3 2739560

[B34] Merriam J. J. Mathur R. Maxfield-Boumil R. Isberg R. R. (1997). Analysis of the *Legionella pneumophila fliI* gene: intracellular growth of a defined mutant defective for flagellum biosynthesis. Infect. Immun. 65, 2497–2501. 9169800 10.1128/iai.65.6.2497-2501.1997 PMC175352

[B35] Miller H. K. Kwuan L. Schwiesow L. Bernick D. L. Mettert E. Ramirez H. A.. (2014). IscR is essential for *Yersinia pseudotuberculosis* type III secretion and virulence. PLoS Pathog. 10:e1004194. 10.1371/journal.ppat.1004194 24945271 PMC4055776

[B36] Morgan J. M. Duncan M. C. Johnson K. S. Diepold A. Lam H. Dupzyk A. J.. (2017). Piericidin A1 blocks *Yersinia* ysc type III secretion system needle assembly. mSphere 2:e00030–17. 10.1128/mSphere.00030-17 28217742 PMC5311113

[B37] Negrea A. Bjur E. Ygberg S. E. Elofsson M. Wolf-Watz H. Rhen M. (2007). Salicylidene acylhydrazides that affect type III protein secretion in *Salmonella enterica serovar typhimurium*. Antimicrob. Agents Chemother. 51, 2867–2876. 10.1128/AAC.00223-07 17548496 PMC1932493

[B38] Nordfelth R. Kauppi A. M. Norberg H. A. Wolf-Watz H. Elofsson M. (2005). Small-molecule inhibitors specifically targeting type III secretion. Infect. Immun. 73, 3104–3114. 10.1128/IAI.73.5.3104-3114.2005 15845518 PMC1087345

[B39] O'Callaghan C. H. Morris A. Kirby S. M. Shingler A. H. (1972). Novel method for detection of beta-lactamases by using a chromogenic cephalosporin substrate. Antimicrob. Agents Chemother. 1, 283–288. 10.1128/AAC.1.4.283 4208895 PMC444209

[B40] Perry R. D. Harmon P. A. Bowmer W. S. Straley S. C. (1986). A low-Ca^2+^ response operon encodes the V antigen of *Yersinia pestis*. Infect. Immun. 54, 428–434. 3021629 10.1128/iai.54.2.428-434.1986 PMC260179

[B41] Pettersson J. Nordfelth R. Dubinina E. Bergman T. Gustafsson M. Magnusson K. E.. (1996). Modulation of virulence factor expression by pathogen target cell contact. Science 273, 1231–1233. 8703058 10.1126/science.273.5279.1231

[B42] Portnoy D. A. Moseley S. L. Falkow S. (1981). Characterization of plasmids and plasmid-associated determinants of *Yersinia enterocolitica* pathogenesis. Infect. Immun. 31, 775–782. 7216474 10.1128/iai.31.2.775-782.1981 PMC351377

[B43] Rosqvist R. Magnusson K. E. Wolf-Watz H. (1994). Target cell contact triggers expression and polarized transfer of *Yersinia* yopE cytotoxin into mammalian cells. EMBO J. 13, 964–972. 8112310 10.1002/j.1460-2075.1994.tb06341.x PMC394898

[B44] Sample A. K. Fowler J. M. Brubaker R. R. (1987). Modulation of the low-calcium response in *Yersinia pestis* via plasmid-plasmid interaction. Microb. Pathog. 2, 443–453. 10.1016/0882-4010(87)90051-9 3507558

[B45] Schwiesow L. Lam H. Dersch P. Auerbuch V. (2015). *Yersinia* type III secretion system master regulator LcrF. J. Bacteriol. 198, 604–614. 10.1128/JB.00686-15 26644429 PMC4751813

[B46] Skinner S. O. Sepúlveda L. A. Xu H. Golding I. (2013). Measuring mRNA copy number in individual *Escherichia coli* cells using single-molecule fluorescent *in situ* hybridization. Nat. Protoc. 8, 1100–1113. 10.1038/nprot.2013.066 23680982 PMC4029592

[B47] Straley S. C. Bowmer W. S. (1986). Virulence genes regulated at the transcriptional level by Ca^2+^ in *Yersinia pestis* include structural genes for outer membrane proteins. Infect. Immun. 51, 445–454. 3002984 10.1128/iai.51.2.445-454.1986 PMC262351

[B48] Tree J. J. Wang D. McInally C. Mahajan A. Layton A. Houghton I.. (2009). Characterization of the effects of salicylidene acylhydrazide compounds on type III secretion in *Escherichia coli* o157:H7. Infect. Immun. 77, 4209–4220. 10.1128/IAI.00562-09 19635828 PMC2747932

[B49] Veenendaal A. K. Sundin C. Blocker A. J. (2009). Small-molecule type III secretion system inhibitors block assembly of the *Shigella* type III secreton. J. Bacteriol. 191, 563–570. 10.1128/JB.01004-08 18996990 PMC2620818

[B50] Wang H. Avican K. Fahlgren A. Erttmann S. F. Nuss A. M. Dersch P.. (2016). Increased plasmid copy number is essential for *Yersinia* T3SS function and virulence. Science 353, 492–495. 10.1126/science.aaf7501 27365311

[B51] Wilharm G. Lehmann V. Krauss K. Lehnert B. Richter S. Ruckdeschel K. (2004). *Yersinia enterocolitica* type III secretion depends on the proton motive force but not on the flagellar motor components motA and motB. Infect. Immun. 72, 4004–4009. 10.1128/IAI.72.7.4004-4009.2004 15213145 PMC427454

[B52] Yother J. Goguen J. D. (1985). Isolation and characterization of Ca^2+^-blind mutants of *Yersinia pestis*. J. Bacteriol. 164, 704–711. 2997127 10.1128/jb.164.2.704-711.1985 PMC214309

